# Grazing management and stocking strategy decisions for pasture-based beef systems: experimental confirmation vs. testimonials and perceptions

**DOI:** 10.1093/tas/txad069

**Published:** 2023-06-28

**Authors:** Francis Monte Rouquette, Lynn E Sollenberger, João M B Vendramini

**Affiliations:** Soil & Crop Sciences, Texas A&M AgriLife Research, Overton, Texas, USA; University of Florida, Gainesville, Florida, USA; University of Florida – IFAS, Ona, Florida, USA

**Keywords:** experimental evidence, grazing management, mob stocking, pastures, regenerative grazing, rotational stocking

## Abstract

Grazing management and stocking strategy decisions involve the manipulation of grazing intensity, grazing frequency, and timing of grazing to meet specific objectives for pasture sustainability and economic livestock production. Although there are numerous stocking systems used by stakeholders, these methods may be broadly categorized as either continuous or some form of rotational stocking. In approximately 30 published experiments comparing continuous vs. rotational stocking, there was no difference in liveweight gain per animal between stocking methods in 66% of studies. There was no difference in gain per hectare between methods in 69% of studies, although for gain per hectare the choice of fixed or variable stocking rate methodology affected the proportion (92% for fixed; 50% for variable). Despite these experimental results showing limited instances of difference between rotational and continuous stocking, rotational strategies (e.g., “mob stocking” or “regenerative grazing”) have received what appears to be unmerited acclaim for use for livestock production. Many proposed “mob stocking” or “regenerative grazing” systems are based on philosophies similar to high intensity-low frequency stocking, including provision for >60 d of rest period from grazing. In addition, grazing management practitioners and stakeholders have voiced and proposed major positive benefits from rotational stocking, “mob stocking”, or “regenerative grazing” for soil health attributes, carbon sequestration, and ecosystem services, without experimental evidence. The perceptions and testimonials supporting undefined stocking systems and methods have potential to mislead practitioners and result in economic disservices. Thus, we suggest that scientists, extension-industry professionals, and producers seek replicated experimental data as the basis for predicting outcomes of grazing decisions.

## GRAZING MANAGEMENT

Grazing management has been defined as the manipulation of grazing in pursuit of a specific objective or set of objectives (Allen et al., 2011). Those strategies that can be manipulated include grazing intensity, grazing frequency, and timing of grazing (Sollenberger et al., 2020). Sollenberger et al. (2020) characterized grazing intensity as severity of grazing. Grazing intensity is sometimes described using animal-based measures such as stocking rate, or pasture-based measures such as forage mass or plant height. However, these descriptions refer to only one component of the grazing system, i.e., animal or forage; they do not integrate both of these components for purposes of management. Grazing intensity is best described as forage allowance (amount of forage dry matter per unit animal liveweight; Forage DM:Animal BW), or as grazing pressure (relationship between animal body weight and amount of forage; Animal BW:Forage DM); thus, both pasture- and animal-based factors are combined (Sollenberger et al., 2005; Allen et al., 2011). Grazing frequency is related to stocking method and includes continuous stocking through a wide range of rotational stocking methods (Allen et al., 2011). Timing of grazing relates to the physiological stage of forage growth and maturity when grazed, or to the chronological time in the season when grazing occurs.

Whether grazing management strategies are based on experimental evidence, experience, or perceptions-philosophy, management systems may be difficult to change, alter, or amend. Rouquette and Aiken (2020) stated that “forage-based livestock production is challenged to enhance sustainability of pastures and cattle production, and to maintain economic stability in the presence of changes in market prices of cattle, fertilizer, feed, and other requirements. Management strategies that meet production goals while maintaining soil and ecosystem health, including minimal impact on the environment, require a basic understanding of how: 1) the intensity, rate, and duration of stocking will impact cattle performance and production; 2) grazing systems can be used to maintain sustainable, productive pastures; 3) innovations in feeding and watering systems can be used to minimize negative impacts on water and soil health; 4) management of soil nutrients, which affects nutrient cycling, can be effective in minimizing environmental impacts and controlling input costs; 5) control of noxious weeds can be realized in order to maintain forage composition, pasture condition, and ecosystem stability; and 6) forage systems can accommodate wildlife habitat and diet requirements.” Sustainable beef (Global Roundtable for Sustainable Beef, 2016) has been defined as a socially responsible, environmentally sound, and economically viable product that prioritizes natural resources, efficiency and innovation, people and community, animal health and welfare, and food.

### Vegetational-Hardiness Zones and Forages

Grazing management strategies and implementations vary among introduced forages on pastures and native plant species on rangelands. Although management and mindsets may be targeted toward sustainable beef cattle systems, the Vegetational-Hardiness Zones of semi-arid vs. humid conditions determine the best adapted and persistent forages in each region. For example, in the more humid regions, warm-season perennial sod-forming grasses such as bermudagrass [*Cynodon dactylon* (L.) Pers] and bahiagrass (*Paspalum notatum* Flügge) are best adapted and tolerant of increased grazing pressure. These forages may also be harvested for hay, baleage, or silage. Alternatively, in semi-arid regions, native perennial warm-season bunch grasses and other forbs and browse are the best-adapted forages for rangelands. Tolerance to frequency and severity of defoliation regimens differ for sod-forming rhizomatous grasses in humid areas vs. bunch grasses in semi-arid regions. Thus, stocking strategies and expected economic return may be substantially different among grazing-land ecosystems.

### Stocking Methods

Stocking method has been defined as a “procedure or technique to manipulate animals in space and time to achieve a specific objective” (Allen et al., 2011; Table 1). They suggested that the objectives of a specific stocking method could vary from: a) allocating forage nutritive value among livestock classes; b) enhancing efficiency of forage utilization; c) avoiding repeated defoliation of the plant which may lead to decreased forage accumulation and persistence; to d) extending the stocking season. Stocking methods can be categorized as variations of continuous or rotational stocking. Continuous stocking is a method of grazing livestock on a specific unit of land where animals have unrestricted and uninterrupted access throughout the time when grazing is allowed; whereas rotational stocking is a method of grazing livestock that utilizes recurring periods of grazing and rest among three or more paddocks in a grazing management unit throughout the time when grazing is allowed (Allen et al., 2011).

**Table 1.** Some examples of stocking methods defined by Allen et al. (2011)

Alternative stocking	Mob stocking
Continuous Stocking	Nonselective stocking
Creep stocking	Put-and-take stocking
Deferred stocking	Ration stocking
First-last stocking	Rotational stocking
Forward-creep stocking	Seasonal stocking
Frontal stocking	Sequence stocking
Intensive early stocking	Set stocking
Intermittent stocking	Strip stocking
Mixed stocking	Variable stocking

With respect to published experimentation on pastures, Sollenberger et al. (2012) compiled the results of continuous vs. rotational stocking in a review of 19 refereed journal papers. These publications included 29 separate experiments on the comparison of gain per animal responses, and 26 separate comparisons of gain per hectare on continuous vs. rotationally stocked pastures. These experiments were conducted in different states in the United States over a period of more than 20 yr. Data collected included forage mass, nutritive value, gain per animal, stocking rate, and gain per hectare. More than 70% of these experiments showed no effect of stocking method on forage nutritive value. However, nearly 85% of these studies showed an advantage for rotationally stocked pastures in forage mass or pasture carrying capacity (stocking rate). The average increase in forage mass was about 30% for rotationally stocked vs. continuously stocked pastures.

With minimal to no effect of stocking method on nutritive value, but with an increase in forage mass with rotational stocking, how does this translate to gain per animal (ADG) and gain per hectare for various stocking methods? With respect to ADG, 66% of the studies showed no difference between stocking methods; 20% showed continuous to be greater than rotational; and 14% of the studies showed that rotationally had greater ADG than continuously stocked pastures. This conforms to expectations since more than 70% of studies found no effect of stocking method on nutritive value. While some presume that rotational stocking results in greater nutritive value than continuous stocking, there are factors working against this presumption. In rotationally stocked swards, cattle may be subjected to greater grazing intensities to achieve a greater percentage of forage utilization in the resident paddock (Rouquette, 2015). Forced consumption of forage into the lower part of the standing crop (sward) may result in consumption of a greater proportion of the stems which are of lesser nutritive value. Additionally, cattle on continuously stocked pastures with a moderate stocking rate have opportunities for selectivity and can choose immature forage. The period between grazing events (i.e., maturity) on a rotationally stocked pasture is determined largely by the pasture manager which likely leads to consumption of more mature forage on rotationally than continuously stocked pastures (Sollenberger et al., 2012). When given a choice, cattle select more than 80% of their diet as leaves (Roth et al., 1990). Thus, greater diet nutritive value can occur with continuous vs. rotational stocking, specifically when pastures are not overstocked.

Forage mass and forage allowance set the boundaries for potential ADG. However, forage nutritive value is responsible for setting the upper limits on ADG (Sollenberger and Vanzant, 2011). Therefore, both forage mass and nutritive value are collectively responsible for attaining maximum ADG from pastures (Rouquette, 2015). What about gain per hectare and stocking methods? In the Sollenberger et al. (2012) review of 26 grazing experiments that reported results for gain per hectare, 69% of the studies showed no difference between continuous vs. rotational stocking; 27% showed an advantage for rotational stocking (all on cool-season forages); and 4% showed an advantage for continuous stocking (all on ‘Coastal’ bermudagrass). Methodological issues exist in interpreting these results because some of the studies used a fixed stocking rate for comparing stocking methods. When stocking rate is fixed, there is no opportunity for the documented greater carrying capacity of rotationally stocked pastures (Sollenberger et al., 2012) to be demonstrated, thus minimizing the likelihood of a gain per hectare response to stocking method. In fact, when the literature assessment considered only experiments where a variable stocking rate was used in the stocking method comparison, the proportion of experiments where rotational stocking had greater gain per hectare rose to 50% (from 27%), compared with 7% (from 4%) where continuous had greater gain per hectare, and reduced to 43% (from 69%) for no difference. Thus, the variable stocking rate approach is preferred for comparisons of animal response to stocking method because it allows for the increased forage mass advantage of rotationally stocked pastures to translate into greater stocking rate, and about half the time into greater gain per hectare.

In addition to the potential impact on forage and animal performance, Dubeux et al. (2014) observed that stocking method may affect excreta distribution, consequently affecting soil nutrient distribution in grazed bahiagrass swards. Rotational stocking with a 1-d grazing period had a more uniform dung distribution than a 7-d grazing period and a continuous stocking treatment, which resulted in less spatial variability in P, K, and Mg soil concentrations. Matthews et al. (1999) hypothesized that greater stocking densities due to subdivision of the pasture would cause greater competition for forage, and the animals would spend less time under the shade and more time close to the water source. They found, however, that in warm climates or during periods of hot weather in temperate climates, shade and water sources may have a greater influence on animal behavior than stocking method, thus reducing the likelihood of differences among methods (Matthews et al., 1994).

### What is Best for Pastures: Continuous or Rotational?

“Few topics in agriculture have been addressed with such charismatic language and with such abandonment of scientific evidence and logic” as discussions of continuous vs. rotational stocking (Bransby 1988, 1991). In many stocking method discussions, the debates tend to be driven by testimonials and perceptions instead of experimental data. The stocking method of choice eventually becomes a personal decision for management and may not have been assessed as the “best method”. Selecting management and stocking strategies to make optimum use of forage production, individual animal performance, and overall gain per unit land area led to the concept of Flexible Grazing Systems (Blaser et al., 1962). These grazing systems may not be “hardcore rotationally, time-scheduled stocked”, but they do involve multiple pastures with strategies to incorporate flexible movement of cattle based on forage needs for grazing and deferred forages in concert with desired ADG for economic returns per unit land area. Some examples of these flexible stocking strategies include: a) systems for fattening steers on pasture (Blaser et al.,1956); b) creep or forward-creep grazing (Blaser et al., 1986); c) a two-herd system of first and last grazers (Rouquette et al., 1992); and d) a three-herd system using different classes of cattle (Rouquette et al.,1994).

### Rangeland: Continuous vs. Rotational Stocking

Briske et al. (2008) reviewed experiments comparing continuous vs. rotational stocking of rangeland. Although rotational stocking was a viable stocking method for rangeland, the perception that rotational was superior to continuous stocking was not supported by the majority of experimental investigations. They further concluded that the continued advocacy for rotational stocking as a superior system for rangeland was based on perception and anecdotal interpretations rather than on experimental results. Briske et al. (2014) described holistic grazing as successive periods of grazing by livestock that are concentrated in a single herd to produce a high grazing pressure and followed by rest periods when supported by adaptive management and holistic management. They conducted an assessment of holistic management for rangeland and concluded that “the vast majority of experimental evidence does not support claims of enhanced ecological benefits in intensive rotational grazing compared to other stocking strategies, including the capacity to increase storage of soil organic carbon”. They concluded that of all the practices one may adopt for grazing, stocking rate is the primary factor that controls the resultant sustainability of rangeland as a forage source. Carter et al. (2014) reported peer-reviewed studies that showed holistic management of intensive rotational stocking with a single herd for short durations with long rest periods was not superior to conventional grazing systems. They further suggested that stocking rate, rest period, and livestock exclusion may represent the best management strategies for restoring native grassland productivity, ecological condition, and sustainability. Hawkins (2017) reported that holistic management is a goal-setting process that may be used to define quality of life, form of production, and a future resource base for the landowner. It was concluded from peer-reviewed publications that holistic management, also termed holistic planned grazing, does not improve production; thus, this management approach does not warrant the additional inputs of infrastructure and labor. Hawkins (2017) also reported that other terminology for holistic management may include holistic resource management or the Savory (Savory, 1983) method.

### Mob Stocking

Allen et al. (2011) defined mob stocking as “a method of stocking at a high grazing pressure for a short time to remove forage rapidly as a management strategy”. What is a mob? How are livestock mobs controlled? One of the first uses of the term “mob stocking” has been attributed to G.O. Mott after visiting with Australian researchers (Gurda et al., 2018). The terminology and application were first used in evaluation of warm-season perennial grass cultivars and germplasm (Mislevy et al., 1983; Rouquette and Florence, 1983; Gildersleeve et al., 1987). Within these studies, management treatments (i.e., interval between grazing events, sward canopy height at the end of a grazing event) varied among species and experiments, with the common feature being a high stocking density imposed for a short period of time on small experimental units. This approach allowed researchers to test a greater number of treatments under grazing conditions, when only pasture responses were being measured. It is important to note that neither in the definition of mob stocking (Allen et al., 2011) nor in its early use in forage evaluation was there any implication that long rest periods between defoliation events were a required part of this technique. This is a more recent “add on” to the definition and does not conform to the classic definition of mob stocking or the manner in which it was initially practiced. From these initial uses as a defoliation technique in small experimental units for forage cultivar evaluation, mob stocking has been promoted as a viable, economic, and biological enhancement strategy for grazing management on a commercial scale. This promotion has not been accompanied by experimental evidence supporting its benefits or defining optimum grazing practices including interval between grazing events, stocking rate or sward height, etc. Nonreplicated demonstrations in semi-arid or humid environments generally have not used or yielded well-defined management criteria, leaving a void of comparative data to describe recommended grazing practices or effects on ecosystem services, including soil health attributes.

Some studies comparing various mob-stocking approaches with more conventional grazing practices have been conducted. A multiyear and replicated mob-stocking experiment was conducted in the Nebraska sandhills (Redden, 2014; Lindsey, 2016; Andrade et al., 2022). During an approximately 75-d period in each of 8 yr, a 120-paddock rotational system with one grazing event annually was compared to a four-paddock rotational stocking system with either one or two grazing events. The ADG of yearling steers at the same stocking rate was different for each method at approximately 0.18 kg/d for the 120-paddock system, 0.57 kg/d for the four paddock system with one grazing event, and approximately 1.0 kg/d for the four paddock with two grazing events. The overall summary from this multiyear, replicated experiment was that there were no effects of stocking method on plant species composition, forage mass, or root growth dynamics. The authors concluded that the additional infrastructure, labor, and management costs of the 120-paddock system could not be justified in this vegetational area.

Mob, rotational, and continuous stocking were evaluated in a temperate grassland area with endophyte-infected tall fescue (*Festuca arundinacea* Schreb), orchardgrass (*Dactylis glomerate* L.), Kentucky bluegrass (*Poa pratensis* L.), white clover (*Trifolium repens* L.), and red clover (*Trifolium pratense* L.) during 3 yr in Virginia (Tracy and Bauer, 2019). Forage mass and nutritive value were similar across all stocking methods. Cow-calf performance was reduced under mob stocking. They concluded that mob stocking may be a beneficial strategy for short-term vegetation management rather than for season-long stocking. In addition, the authors suggested that mob stocking as practiced in this study was an unwise investment due to the limited benefits for both forage and livestock.

Mob stocking may offer benefits in environments and conditions with a diverse, multispecies forage and browse vegetation, keeping in mind that the recent construct of mob stocking requiring long intervals between defoliation events is not part of the accepted definition of the method. Management should be reminded that if long intervals between grazing events are used, mature, nonlactating cows may be the “best suited” cattle for use due to their relatively low nutrient requirements, which are primarily for maintenance and not for growth, lactation, and/or estrus. The “least desirable” cattle to use in the long rest period iteration of mob stocking are young, lightweight (200 to 300 kg BW) stocker-yearlings due to their high nutrient requirements and the reduced nutritive value of the more mature forage available for selection in successive paddocks.

## EXPERIMENTAL EVIDENCE OR TESTIMONIALS

Stocking method terminology has taken on a multitude of “catch phrases” that may have individual or locational meaning; however, these terms often add to confusion among scientists and stakeholders. Castillo and Wallau (2023) reviewed stocking terminology that has been used in educational, outreach, and engagement programs. During the past several years, some of the following terminology has arisen and infers a form of rotational stocking, including: a) intensive grazing; b) management intensive grazing; c) adaptive multipaddock grazing; d) adaptive grazing management; e) flash grazing; f) flexible grazing; and g) regenerative grazing. Many of these stocking methods have been recommended and promoted without specific definition and thus lack comparative data with continuous or other rotational stocking methods. Terminology has become the focus of attention rather than data that might provide confirmation of the value of implementation of these methods for animal and pasture performance.

### Is Regenerative Grazing a Mob-Stocking Method?

The word “regenerative” has become popular terminology for sales and promotion of remedies, products, and actions. Today, one can adopt or be engaged with regenerative medicine, health, energy, economics, engineering, agriculture, and grazing. Giller et al. (2021) reviewed the origins of regenerative agriculture and the various philosophies and definitions for its implementation. They found that many producer testimonials on the internet suggested their adoption of regenerative agriculture was “underpinned by the philosophy that seeks to protect and enhance the environment”. They also reported that many regenerative practices such as crop residue retention, cover cropping, and reduced tillage were central to the “canon of good agricultural practices”; whereas other practices were contested and at best were specific to a particular “niche”, such as permaculture and holistic grazing.

Regenerative grazing has been defined, without comparative data or experimental evidence, as stocking practices or methods that enhance ecosystem services, soil health, and soil conservation. The adoption and incorporation of regenerative grazing systems has basically been accepted as a timed, “auto-graze method”. This auto-graze philosophy has been readily accepted primarily because it is a method or system that combines all cattle into a single paddock with timed relocation to the next paddock generally on a daily or twice-daily basis. Thus, this stocking approach allows for managers to “start the process” and then let time and rotations make default management decisions of matching forage production-nutritive value to animal requirements. From the perspective of the novice, as well as from seasoned managers, there is a human behavior tendency to follow popular methods or practices. Thus, there is an increasing adoption of a method or system that has a popular name and substitutes for regular visual inspections of pastures to determine the best management practice for sustainable pastures and sustainable beef systems. This approach often includes intensive rotational stocking that may range from one to a few days’ residence on a pasture with 30 to 60 d or more rest. It also does not conform to accepted definitions of mob stocking, which do not require long periods between defoliation events. Thus, regenerative grazing is not a specific practice like mob stocking, but rather it is based on various philosophies and testimonials of management or strategies that are perceived to promote soil health, carbon sequestration for credit accountability, and sustainability.

### Regenerative or Sustainable Agriculture

Giller et al. (2021) investigated the recent terminology of “regenerative agriculture” from an agronomic perspective. Although this terminology has been in use from the early 1980s, only since 2016 has regenerative agriculture been adopted and incorporated as a “buzz word” used by multinational companies, charitable foundations, USDA, etc. Regenerative agriculture is not significantly different than sustainable agriculture, organic farming, sustainable intensification, climate-smart agriculture, agroecology, etc. Giller et al. (2021) further suggested that academic and research agronomists need to engage constructively with individuals, organizations, and corporations that champion regenerative agriculture and more clearly define the elements of this method through scientific investigation. They provided areas and questions to be addressed that would assess the agronomic aspects of the mechanism and dynamics of regeneration. In summation, they suggested that such investigations “will also help to separate the philosophical baggage and some of the extraordinary claims that are linked to regenerative agriculture, from the areas and problems where agronomic research might make a significant contribution”.

### Stocking Strategies and Sustainability of Pasture-Beef Systems

Rouquette (2015) suggested that managers must consider some of the following factors to optimize output from the system: a) understanding forage growth and regrowth; b) “hands on” experience with animals and animal husbandry; c) intuitive application of decisions for input and outputs; d) knowledge of current and forecasted weather conditions in the specific ecoregion; e) ability to assume the risks associated with stocking outcomes; f) constant awareness of vegetation and land resources; and g) an alternative or “escape plan” for animals in the event of extreme weather conditions.

Stocking strategies should be characterized or designed within a specific vegetation or hardiness zone and combined with the art and science of management for efficient-strategic forage utilization and sustainability for the desired optimum pasture-animal production. Thus, management strategies are site-specific for multiple input–output decisions with objectives to “match” forage-animal requirements to production and economic rewards (Rouquette, 2015). Grazing management should be based on experimental evidence with opportunities to customize strategies to fit within a manager’s level of economic risk and desired-expected environmental stewardship goals. These management strategies should be focused on integrating relationships of pasture ecosystems and stand maintenance, environmental awareness, economic implications, and legacy-heritability objectives of property for sustainable forage-livestock production (Rouquette, 2017).

## CONCLUSIONS

Implementing revised or new management strategies requires attention to detail and the use of data-driven results from comparative experiments. These strategies may include: fertilizer ingredients and rates for hay or pasture; supplementation ingredients and amount for specific classes of livestock; breed type for cow–calf and/or stocker operations; forage cultivars for perennial and/or annual pastures; stocking method for sustainable beef system and economic returns; and seasonal and/or year-long stocking rate or carrying capacity of a particular property. Sollenberger et al. (2020) summarized that “Within the community of grazing management practitioners, proponents of one approach or another may rely too heavily on anecdotes and too lightly on data”. They also suggested that “before adopting a new grazing management approach, there is value in requesting DATA that support the recommendations being made. It is equally important that the source of the data be an independent organization without conflict of interest, and that the experiments be conducted on a time and size scale that provides relevant results to producers”. While grazing management strategies developed under replicated research protocols are available without monetary charge, they may not be readily accepted and implemented because promised outcomes are tempered by reality. At the same time, strategies based on perception and without supporting scientific data may be widely accepted due to promised outcomes that are extremely attractive but may exceed that which is biologically achievable.

The Land Grant System routinely disseminates data through State Agricultural Experiment Stations and Extension Service publications and short courses. Their recommendations-suggestions are based on multiyear and/or multilocational comparative research and experimental data. To better reflect current methods of accessing information, we suggest that forage-animal scientists increase and enhance distribution of grazing systems research results through social media content that is focused, concise, free of scientific jargon, and designed to attract the novice and seasoned manager audience alike. Grazing systems should be viewed as a “work in progress”, as management fine-tunes input strategies and delivery systems for sustainable pasture-livestock production and ecosystem services that benefit soil-water components and provide positive economic returns.

## CNFLICTS OF INTEREST STATEMENT

The authors affirm that they have no affiliations with nor involvement in any organization or entity with any financial interest (such as honoraria; educational grants; membership, employment, consultation, or other equity interest; or expert testimony or patent-licensing arrangements) in the subject matter or materials discussed in this manuscript.
